# A Mitocentric View of the Main Bacterial and Parasitic Infectious Diseases in the Pediatric Population

**DOI:** 10.3390/ijms22063272

**Published:** 2021-03-23

**Authors:** Sonia Romero-Cordero, Richard Kirwan, Antoni Noguera-Julian, Francesc Cardellach, Clàudia Fortuny, Constanza Morén

**Affiliations:** 1 Faculty of Medicine, Pompeu Fabra University and Universitat Autònoma de Barcelona, 08002 Barcelona, Spain; sonia.romero.cordero@gmail.com; 2 School of Biological and Environmental Sciences, Liverpool John Moores University, Liverpool L2 2QP, UK; 3 Malalties Infeccioses i Resposta Inflamatòria Sistèmica en Pediatria, Unitat d’Infeccions, Servei de Pediatria, Institut de Recerca Pediàtrica Hospital Sant Joan de Déu, 08950 Barcelona, Spain; ton@hsjdbcn.org (A.N.-J.); cfortuny@hsjdbcn.org (C.F.); 4 Faculty of Medicine and Health Sciences, University of Barcelona, 08036 Barcelona, Spain; fcardell@clinic.cat; 5 Centro de Investigación Biomédica en Red de Epidemiología y Salud Pública (CIBERESP), 28029 Madrid, Spain; 6 Red de Investigación Translacional en Infectología Pediátrica (RITIP), 28029 Madrid, Spain; 7 Muscle Research and Mitochondrial Function Laboratory, Cellex-IDIBAPS, Faculty of Medicine and Health Sciences, University of Barcelona, 08036 Barcelona, Spain; 8 Centro de Investigación Biomédica en Red de Enfermedades Raras (CIBERER) (ISCIII), 28029 Madrid, Spain; 9 Internal Medicine Department-Hospital Clínic of Barcelona (HCB), 08036 Barcelona, Spain

**Keywords:** antibiotics, infections, mitochondria, pediatrics

## Abstract

Infectious diseases occur worldwide with great frequency in both adults and children. Both infections and their treatments trigger mitochondrial interactions at multiple levels: (i) incorporation of damaged or mutated proteins to the complexes of the electron transport chain, (ii) mitochondrial genome (depletion, deletions, and point mutations) and mitochondrial dynamics (fusion and fission), (iii) membrane potential, (iv) apoptotic regulation, (v) generation of reactive oxygen species, among others. Such alterations may result in serious adverse clinical events with great impact on children’s quality of life, even resulting in death. As such, bacterial agents are frequently associated with loss of mitochondrial membrane potential and cytochrome c release, ultimately leading to mitochondrial apoptosis by activation of caspases-3 and -9. Using Rayyan QCRI software for systematic reviews, we explore the association between mitochondrial alterations and pediatric infections including (i) bacterial: *M. tuberculosis*, *E. cloacae, P. mirabilis, E. coli, S. enterica, S. aureus, S. pneumoniae, N. meningitidis* and (ii) parasitic: *P. falciparum*. We analyze how these pediatric infections and their treatments may lead to mitochondrial deterioration in this especially vulnerable population, with the intention of improving both the understanding of these diseases and their management in clinical practice.

## 1. Introduction

The burden of infectious diseases worldwide has considerable impact resulting in 350,000 deaths in 2017 according to the WHO [1]. As such, fully understanding the main molecular, subclinical events associated with such infections is of vital importance to clinical research. This systematic review focuses on the mitochondrial changes associated with the principal bacterial and parasitic infections, as well as their treatments, in the pediatric population, an especially vulnerable group. Here, we review how both infections and their treatments promote molecular mitochondrial damage at multiple levels, including depletion, deletions, and point mutations in the mitochondrial genome, mutated mitochondrial proteins, and alterations in mitochondrial dynamics, clearance, membrane potential, apoptosis, and oxidative damage. All these molecular events may compromise cell viability, which ultimately may result in serious adverse clinical events, considerably impacting quality of life or even resulting in death. To provide a complete overview of these molecular alterations resulting from the main pediatric bacterial and parasitic infections (including *M. tuberculosis*, *E. cloacae*, *P. mirabilis*, *E. coli*, *S. enterica*, *S. aureus*, *S. pneumoniae*, *N. meningitidis*, and *P. falciparum*), we will first guide the readers through some of the main considerations regarding mitochondrial physiology and pathology, which retain multiple features of their bacterial ancestry, a key issue when considering the mitochondrial effects precisely related to bacterial infections. We will then review, for the first time to our knowledge, the specific mechanisms by which mitochondria are affected by the aforementioned bacterial and parasitic infections, as well as their corresponding treatments in children.

## 2. Mitochondria

**Figure 1:**
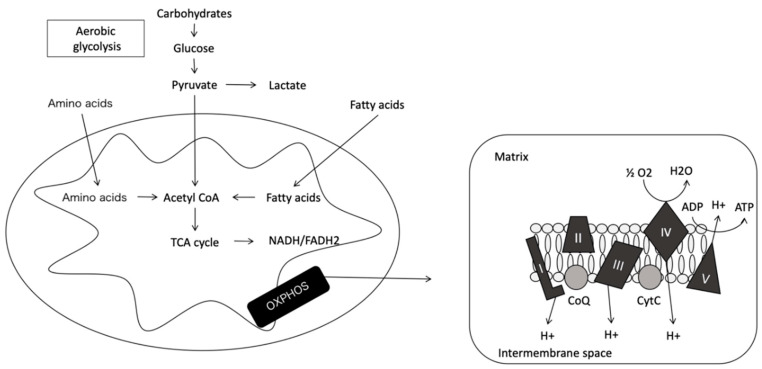
Simplified general summary of the main mitochondrial metabolic pathways. ADP, adenosine diphosphate; ATP, adenosine triphosphate; I, complex I; II, complex II; III, complex III; IV, complex IV; CoQ, coenzyme Q; CytC, cytochrome C; FADH, flavin and adenine dinucleotide; H+, proton; NADH, nicotinamide adenine dinucleotide hydrogen; OXPHOS, oxidative phosphorylation system; TCA, tricarboxylic acid and V, V complex.

Mitochondria are semi-autonomous, maternally inherited organelles present in the cytoplasm of virtually all eukaryotic cells [2,3]. They are essential for cell viability due to their involvement in cellular respiration, apoptosis, catabolism and anabolism of metabolites, calcium homeostasis, heat production, and energy, through the formation of adenosine triphosphate (ATP) molecules [4] (Figure 1). Mitochondria are present in variable numbers within cells depending on the energy requirements of the tissue. The greater the energy demand, the greater the number of mitochondria, with the greatest numbers reported in nervous and muscular tissue [5]. With respect to this review, the endosymbiotic theory of Lynn Margulis, claiming a common bacterial and mitochondrial origin [6], deserves special attention and is supported by the shared characteristics of both the microorganisms and the organelle, many of which are detailed below. 

### 2.1. Mitochondrial Structure

Mitochondria are not static structures within cells; but dynamic, capable of fusion and fission. They consist of:

#### 2.1.1. Outer Mitochondrial Membrane (OMM)

Permeable to ions, metabolites, and polypeptides, due to porins and/or voltage-dependent channels [3].

#### 2.1.2. Inner Mitochondrial Membrane (IMM)

**Figure 2:**
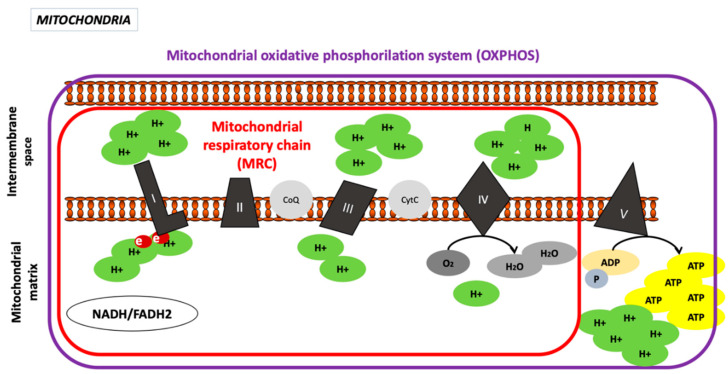
Mitochondrial respiratory chain and oxidative phosphorylation system in the mitochondria. ADP, adenosine diphosphate; ATP, adenosine triphosphate; I, complex I; II, complex II; III, complex III; IV, complex IV; V, complex V; CoQ, coenzyme Q; CytC, cytochrome C; e, electrons; FADH, flavin and adenine dinucleotide; H+, proton; NADH, nicotinamide adenine dinucleotide hydrogen and OXPHOS, oxidative phosphorylation system.

Impervious to almost all molecules and ions, highly selective and rich in cardiolipin. Cardiolipin is a phospholipid found exclusively in the bacterial membrane or in the IMM of eukaryotic cells and represents 10–20% of the total phospholipid content. Transmembrane transport is carried out using proteins specific to certain molecules such as: pyruvic acid, adenosine diphosphate (ADP), ATP, oxygen, water, and fatty acids. Of particular note are adenine nucleotide translocase (ANT), which transports cytosolic ADP to the mitochondrial matrix and ATP (once synthesized) to the cytosol and phosphate translocase, which transfers cytosolic phosphate plus a proton (H+) to the matrix. This phosphate is essential for the phosphorylation of ADP in the oxidative phosphorylation (OXPHOS) process. The IMM presents many folds or invaginations, called mitochondrial cristae, that greatly extend the surface where the enzymatic complexes of the OXPHOS process are embedded [5] (Figure 2).

##### Oxidative Phosphorylation System

Oxidative phosphorylation is the process of ATP synthesis coupled with oxygen consumption, whereby electrons are transferred in stages, through 4 enzymatic complexes (complex I or CI, CII, CIII, and CIV), 2 carriers of mobile electrons (coenzyme Q (CoQ) and cytochrome C (CytC)), which make up the mitochondrial respiratory chain (MRC), and a fifth complex, called ATP synthase or complex V (CV) [3].

In this process, oxygen is consumed and an electrochemical gradient is established, driving ATP synthesis [7]. The electrons flow through the MRC through oxidation–reduction (or redox) reactions ending in complex IV, where oxygen is the final receptor for the electrons and is reduced to H_2_O:

ADP (matrix) + inorganic phosphate (Pi) (matrix) + 3H^+^ (intermembrane) → 2ATP (matrix) + H_2_O + 3H^+^ (matrix)

##### Complex I, Nicotinamide Adenine Dinucleotide Hydrogen (NADH) Dehydrogenase or NADH^−^CoQ Reductase (CI)

It contains flavin mononucleotide (FMN) and transfers electrons to CoQ or ubiquinone:

NADH + H^+^ + CoQ + 4H^+^ (matrix) → NAD^+^ + CoQH_2_ + 4H^+^ (intermembrane space)

The goal is to oxidize the NADH, obtained through the Krebs cycle in the mitochondrial matrix, and transfer the electrons to reduce CoQ. The plant-based insecticide rotenone and the antibiotic piericidin A are specific inhibitors of CI [5].

##### Complex II, Succinate Dehydrogenase or Succinate-CoQ Reductase (CII)

The smallest complex, composed of 4 peptides. The 2 largest peptides make up the peripheral portion of the complex and function as the enzyme “succinate dehydrogenase” in the Krebs cycle. The electrons from the oxidation of succinate to fumarate are channeled through this complex to ubiquinone. Therefore, complex II links the Krebs cycle directly to the MRC. Cofactors, cytochrome b558, and metal ions also constitute CII. A flavin is covalently linked to the largest peptide, producing the flavoprotein subunit (Fp). The overall reaction catalyzed by CII is as follows

Succinate + CoQ → fumarate + CoQH_2_.

It should be noted that in this complex, there is a lack of proton pumping, which takes place at the CI, CIII, and CIV levels. CoQ acts as a mobile carrier of electrons between complexes and receives electrons from CI and CII and transfer them to CIII. The analogue substrate malonate is a specific CII inhibitor, which binds competitively and specifically at the active site in the Fp subunit of the complex [3,5]. 

##### Complex III or CoQH_2_, Known as Cytochrome c Reductase 

Composed of some cytochromes (Cyt b562, Cyt b566, and Cyt c1), as well as an iron–sulfur (Fe-S) group, and transfers electrons from CoQH_2_ to CytC:

CoQH_2_ + 2CytC^3+^ + 2H^+^ (matrix) → CoQ + 2CytC^2+^ + 4H^+^ (intermembrane space).

CytC is a mobile electron carrier protein located on the outer face of the IMM. Its function is to transfer the electrons from the CIII to the CIV [3,5]. Antibiotic antimycin A is a CIII specific inhibitor [5].

##### Complex IV or Cytochrome C Oxidase (COX) 

Transfers electrons to an oxygen molecule reducing it to two water molecules

4CytC_2_^+^ + 8H^+^ + O_2_ → 4CytC_3_+ + 4H^+^ + 2H_2_O.

Molecular oxygen is the terminal electron receptor, the CytC mobile carrier is re-oxidized, and two protons are transferred to the intermembrane space. Potassium cyanide (KCN) is the specific inhibitor of CIV [5].

##### Complex V or ATP Synthase Complex 

Responsible for ATP synthesis through phosphorylation of the ADP molecule. This endergonic reaction is coupled to redox reactions, and electron transport is driven by a proton gradient and constitutes the OXPHOS system. The CV presents two functional subunits: F0 and F1. F0 is a hydrophobic structure and contains a transmembrane channel where the protons are found, enabling the passage from the intermembrane space to the mitochondrial matrix. F1 contains catalytic synthase activity and is composed of five polypeptide chains (α, β, Ɣ, δ, and ε). 

The general reaction that takes place in this complex is:

ADP (matrix) + Pi (matrix) + 3H^+^ (intermembrane) → 2ATP (matrix) + H_2_O + 3H^+^ (matrix).

The ATP produced is the energy required to power all energy-consuming cellular processes [2]. The antibiotic oligomycin is a highly specific inhibitor of the CV F0 subunit.

#### 2.1.3. Intermembrane Space

The space between the OMM and IMM with a similar composition to that of the cytosol and a high concentration of protons as a result of the pumping carried out by the MRC [5]. 

#### 2.1.4. Mitochondrial Matrix 

Contains ions, oxidizable metabolites, and the genetic material of the mitochondria. Along with chloroplasts in the plant cell, mitochondria are the only organelles that contain their own deoxyribonucleic acid (DNA) or mitochondrial DNA (mtDNA) and an autonomous transcriptional and translational capacity, which encodes for some proteins of the OXPHOS [5]. mtDNA lacks introns and does not follow the universal genetic code. Most of the mitochondrial proteins are encoded by nuclear genes and imported to the mitochondria [8] and follow the usual Mendelian inheritance patterns [7]. The mitochondrial genome is found in the matrix as a double-stranded circular DNA, similar to a bacterium’s genome, and synthesizes 2 ribosomal ribonucleic acids (rRNAs), 22 transfer ribonucleic acids (tRNAs), and 13 mRNA that will constitute the MRC subunits. mtDNA can present with heteroplasmy, which is the coexistence of mutated and wild-type mitochondrial genome molecules. This is due to the random distribution that occurs when these organelles divide [5], as mtDNA is randomly distributed into daughter cells by mitotic segregation during cell division [3,5,7].

### 2.2. Mitochondrial Physiology

Mitochondrial functions respond to a series of genetic, metabolic, and neuroendocrine signals with functional and morphological changes and, in turn, generate signals that influence a large number of cellular functions that contribute to the complexity of physiology and pathology. This places the mitochondria in a privileged position, as a “portal” at the intersection of the cell and its environment [8]. Thus, mitochondria have been implicated in aging, regulation of cell metabolism, control of the cell cycle, cell development, defense responses to infections and signal transduction, among other processes [9].

The tricarboxylic acid (TCA) cycle, also called the Krebs cycle or citric acid cycle, which takes place within the matrix of the mitochondria, is a series of eight enzymatic steps that consumes and then regenerates, citrate. It links the metabolism of carbohydrates, fats, and proteins, since the catabolism of these compounds generates acetyl CoA. This key molecule enters the TCA cycle and oxidizes and produces flavin and adenine dinucleotide hydrogen (FADH) and NADH, reducing molecules that feed MRC and OXPHOS [7].

### 2.3. Mitochondrial Pathology

The first patient with mitochondrial disease was described in 1962 [10]. Human mitochondrial diseases are actually a very large collection of hundreds of very heterogeneous and rare diseases, since changes in literally thousands of genes can affect mitochondrial function [7]. Hence, mitochondrial research is on the rise in the medical sciences. As evidence, the number of medical publications related to mitochondriopathies has surpassed those related to other alterations in other organelles, including the endoplasmic reticulum, the Golgi apparatus, and the nucleus [8]. Mitochondrial disorders represent a major challenge in medicine [10]. In the same way, the origin of pleiotropic and multisystemic symptoms in mitochondrial disorders is still poorly understood and often makes it difficult to diagnose this type of disease [8]. Thus, oxidative tissues, with high energy demand (brain, muscle, retina, cochlea, liver, and kidney) are the most vulnerable to OXPHOS defects [10].

Clinical presentations in childhood include allergy, hypotonia, development of mental retardation, conduction failure, seizures, cardiomyopathy, hearing or visual impairment, movement disorders, and lactic acidosis [11].

#### 2.3.1. Anaerobiosis

**Figure 3:**
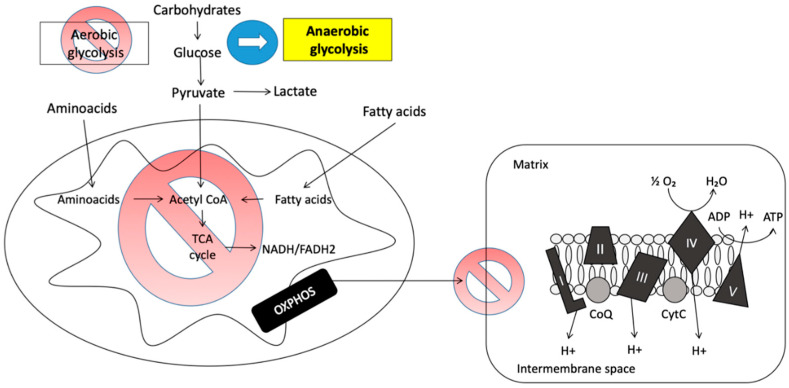
Mitochondrial anaerobiosis state during mitochondrial dysfunction. ADP, adenosine diphosphate; ATP, adenosine triphosphate; I, complex I; II, complex II; III, complex III; IV, complex IV; V, complex V; CoQ, coenzyme Q; CytC, cytochrome C; FADH, flavin and adenine dinucleotide hydrogen; H+, proton; NADH, nicotinamide adenine dinucleotide hydrogen; OXPHOS, oxidative phosphorylation system; TCA, tricarboxylic acid.

In abnormal conditions, such as hypoxia or alterations in mitochondrial function, the metabolic pathways are readjusted to continue obtaining the reducing power necessary for energy production via an anaerobic process (Figure 3). Under these conditions, the pyruvate resulting from catabolism of the metabolites is not imported into the mitochondria, but is converted to lactate through the enzyme lactate dehydrogenase [5]. 

In this pathological context, lactate concentration increases in the blood stream, which comes from its synthesis in skeletal, liver, nervous, and lymphoid tissues. Lactate concentrations under normal conditions range from 0.5 to 2.4 mmol/L [5]. Under conditions of hyperlactatemia, the blood pH falls and acidification occurs [5]. Interestingly, a recent study reported increased lactate in the cerebrospinal fluid of children suffering from meningitis and this may serve as a biomarker to distinguish the bacterial or viral origin of this pediatric infection [12].

#### 2.3.2. Reactive Oxygen Species

Reactive oxygen species (ROS) are intermediate metabolites derived from oxygen generated in the mitochondria during OXPHOS dysfunction. ROS are highly oxidizing free radicals with decoupled electrons that can damage cellular structures, such as proteins, lipids, carbohydrates, genetic material, and also mitochondria, which are particularly vulnerable. ROS are derived from O_2_^−^ (in the MRC at CI and CIII) and include superoxide anion (O_2_^−^), hydrogen peroxide (H_2_O_2_), hydroxyl anion (OH^−^), and peroxynitrite (ONOO^−^) [5]. There are many antioxidants that counteract the deleterious effects of ROS, such as superoxide dismutase (SOD), catalase or peroxidase, and glutathione peroxidase. In addition, there are many non-enzymatic antioxidants, molecules such as vitamins E and C, carotenes, quinones, glutathione, and other metallic elements, such as selenium, zinc, iron, or copper, among others [13]. All of them are capable of reducing ROS levels. Under normal conditions, all antioxidant mechanisms minimize ROS production and therefore, act as a protective system against oxidative stress. However, in the presence of mitochondrial dysfunction, ROS increase beyond the detoxification threshold. Mitochondria and ROS are thus a nexus of multiple pathways that determine the response of cells to disruptions in cellular homeostasis such as infection [14]. Specifically, ROS release may be associated with the presence of exogenous toxic compounds affecting the mitochondria. Nitric oxide (NO) and reactive oxygen species (ROS) produced during bacterial infections are involved critically in host defense mechanisms [15].

#### 2.3.3. Apoptosis

**Figure 4:**
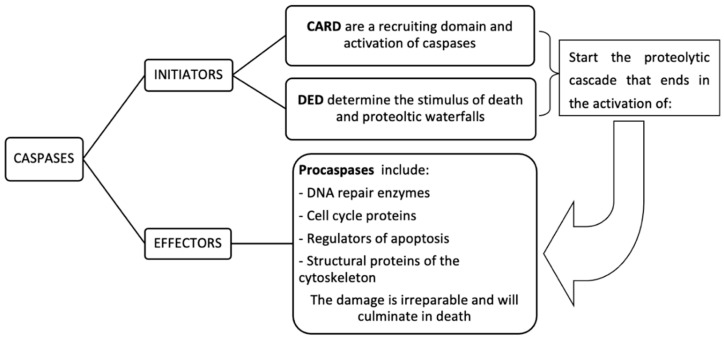
Types of caspases: classification and main functions. Initiator caspases, including CARD [17] and DED [18], and effector caspases, including procaspases, as well as their functions are represented. CARD, caspase activation and recruitment domain; DED, death effector domain.

Apoptosis is a naturally occurring programmed cell death mechanism, which occurs through extrinsic (external trigger) and the intrinsic (internal trigger) pathways and is mainly driven by caspases (serine proteases). Mitochondria play a central role, especially in the intrinsic pathway [16]. Depending on the site of action, apoptotic caspases can be classified into different groups (Figure 4):

In the extrinsic pathway, the stimulus can be external, received by a cell surface receptor. The binding of the apoptosis-inducing ligand to the corresponding receptor leads to the activation of caspase-8 in the cytosol, which, in turn, activates the proapoptotic protein Bid by proteolysis. In this case, mitochondrial involvement in extrinsic apoptosis enhances activation. In the intrinsic pathway, regulated by the mitochondria, the stimulus is internal, as it is the result of the action of a drug, toxin, radiation damage, food shortage or, in general, a situation of stress.

Of course, drugs, toxins, radiation, etc. are also ultimately external influences, but the difference between these two mechanisms, extrinsic and intrinsic, becomes apparent during the next phase, i.e., signal transduction [16]. Active and healthy mitochondria exhibit a mitochondrial transmembrane potential (Δψm) through the IMM where an electrochemical and pH gradient is established as a result of electron transport through the electron transport chain (ETC), with an excess of positive charge in the intermembrane space. In cells undergoing apoptosis, a drop in Δψm is observed as one of the relatively early events, in many different cell types, prior to DNA fragmentation. When induction of the apoptotic process occurs, there are changes in the ratio of proapoptotic (e.g., Bax, Bak, t-Bid, Bim, Bad, and Bik) and antiapoptotic (e.g., bcl-2) proteins. This relationship regulates the permeability of the OMM and its imbalance. Subsequently, the opening of a permeability transition pore (PTP) occurs. If a massive opening of such pores occurs, it can collapse the Δψm, stop OXPHOS, stop the importation of proteins into the mitochondria, and induce leaks of CytC and other apoptogenic mitochondrial proteins, such as SMAC-DEVIL, which exit into the cytosol. On the other hand, mitochondria release an apoptogenic protein, apoptosis-inducing factor (AIF), an intermembrane protein with protease activity but without nuclease activity. CytC binds the cytosolic protein apoptosis protease-activating factor-1 (APAF-1) which, in turn, interacts with procaspase-9 to form a multiprotein complex called the apoptosome. The apoptosome induces the activation of caspase-9 by proteolysis, and caspase-9 activates the caspase-3 effector. This induces irreversible activation of a caspase-catalyzed cascade of reactions and ultimately, deoxyribonuclease (DNAse) is activated by a caspase and cleaves DNA leading to cell death [5]. Mitochondrial alterations can be classified into primary or genetic, when the origin is a genetic alteration, which affects a mitochondrial protein, or secondary or acquired, when the cause is external or environmental, e.g., the bacterial infections [19] and their corresponding antibiotic treatments [20].

Here, we will review those bacterial and parasitic infections and treatments that are associated with mitochondrial involvement and that can be considered of relevance in the pediatric population, with a focus on improving both the understanding of these diseases and their management in clinical practice.

## 3. Bacterial Infectious Processes and Mitochondrial Involvement

### 3.1. Tuberculosis (TB)

TB is a contagious infectious disease caused mainly by *Mycobacterium tuberculosis* (*Mtb*) in humans, which represents one of the top 10 causes of death worldwide and the leading cause of death from a single infectious agent (ranking above HIV/AIDS) [21]. Almost 9 million people present active TB and 2 million die from this disease every year [22,23]. Nonetheless, the probability of developing TB is much higher among people presenting risk factors such as HIV, under-nutrition, diabetes, smoking, and alcohol consumption and also in young children [21]. These groups are also at higher risk of severe and disseminated forms of the disease. 

*Mtb* is spread essentially through the air and enters the human organism mainly through the respiratory route after inhalation. These volatile particles are small enough to reach the lower airways. Infection and the development of the pulmonary form of TB (lungs are the main target of this bacterium) proceed as follows: (i) phagocytosis of the bacilli, (ii) intracellular multiplication, (iii) a latent contained phase of infection, and (iv) active lung infection. These steps can progress towards spontaneous cure, development of disease, latent TB infection and later re-activation, or re-infection [24]. Although the pathogen typically affects the lungs (pulmonary TB), it can also affect other sites (extrapulmonary TB) [21]. 

Symptoms of TB in other parts of the body depend on the area affected. TB in the lungs may cause symptoms such as a chronic cough, chest pain, and hemoptysis. Other unspecific symptoms of TB are weakness or fatigue, weight loss, loss of appetite, chills, fever, and night sweats [24].

#### 3.1.1. Structure and Replication Cycle

*Mtb* infects the alveolar macrophage, through the deposition of infected aerosols in the pulmonary alveoli [25,26]. Each alveolus presents its own alveolar macrophages that are dedicated to constantly cleaning this space [27,28,29,30]. The function of alveolar macrophages is to keep the alveolus clean to allow gas exchange and to avoid any inflammatory development at all costs. The alveolar space is drained with a surfactant generated by pneumocytes, which entails a negative counterpart, and prevents the entry of antibodies [31].

The viable bacillus is phagocytosed by an alveolar macrophage, and then it displays its pathogenic capacity by secreting a 6 kDa early secretory antigenic target (ESAT-6). This peptide is essential to (i) prevent phagosome-lysosome binding, (ii) prevent apoptosis, and (iii) finally, to allow the entry of the bacillus into the cytoplasm. Therefore, the bacillus takes full advantage of its multiplication capacity in a single alveolar macrophage, approximately between 5 and 6 division cycles, to achieve a concentration of between 32 and 64 bacilli per cell. This process takes place for approximately 5–6 days, considering that each division cycle in *Mtb* requires approximately 24 h, causing alveolar macrophage necrosis. To control *Mtb* replication, alveolar macrophages attempt to initiate apoptosis [31]. However, virulent *Mtb* strains are capable of triggering a necrosis-like cell death that is associated with higher mycobacterial replication, strong inflammation, dissemination, and disease progression. Intracellular replication of *Mtb* plays a critical role in determining the fate of the infected cell. If >25 bacilli per cell are achieved, the host cell undergoes an atypical, necrotic-like cell death characterized by lysosomal permeabilization, mitochondrial damage, and membrane destruction. In contrast, macrophages with a low bacillary burden undergo apoptosis, leading to a more advantageous outcome for the host [31].

As the infective process evolves, thousands of bacilli are generated, promoting the synthesis of enough chemokines by infected alveolar macrophages to trigger an inflammatory response [25,32]. With inflammation, there is an imbalance due to an exudate that destroys the alveolar tension and allows the entry of polymorphonuclear cells, normally neutrophils and monocytes, in differential proportions depending on the type of chemokines and cytokines secreted. At the same time, there is more vigorous lavage of the affected alveoli, draining into the lymph nodes through the afferent lymphatic capillaries. In this way, *Mtb* infects the macrophages of the nodules, generating lymphadenitis and dendritic cells [25,33]. As hypersensitivity develops, the inflammatory response becomes more intense, and regional lymph nodes often enlarge. The parenchymal portion of the primary infectious complex is often completely cured by fibrosis or calcification after caseous necrosis and encapsulation. 

The tubercle bacilli of the primary infectious complex can spread through the bloodstream and lymphatic vessels to many parts of the body leading to the development of parenchymal injury and accelerated cassation caused by the development of hypersensitivity [34]. *Mtb* can potentially colonize any organ, and recolonize previously generated lesions that, being in an inflammatory process, have a greater vascularity and permeability, and can also pass into the venous capillaries, reach the left atrium and ventricle, and spread systemically [25,33]. The organs in which endothelial cells allow greater permeability, such as bone tissue, especially in developing children, or the kidney, are common target organs. This spread can involve large numbers of bacilli, leading to disseminated (miliary) TB, or small numbers of bacilli that leave microscopic tuberculous foci scattered in various tissues. These metastatic foci are not clinically apparent initially but are the origin of extrapulmonary TB and pulmonary TB reactivation in some children and in many adults. Extrapulmonary TB represents approximately 30% of cases of TB and usually denotes a delay in the immune response, mainly affecting children under 5 years of age or people suffering from immunosuppression, although there is significant geographic variability, a fact that can be interpreted as the possible existence of some genetic factor that favors it [25,35].

#### 3.1.2. TB in the Pediatric Population

One million children develop TB each year, and 210,000 die from complications of the disease. Increased case rates of childhood TB have been associated with a simultaneous increase in TB rates among HIV-infected adults in the community. Childhood TB is very different from adult TB in epidemiology and clinical and radiographic presentation [36] (Table 1).

A fairly predictable timetable for primary TB and its complications in infants and children is apparent (34). The incubation period in children between the time the Mtb enters the body and the development of cutaneous hypersensitivity is usually 2–12 weeks and most often 4–8 weeks. The onset of hypersensitivity may be accompanied by a febrile reaction that lasts from 1 to 3 weeks. During this phase of intensified tissue reaction, the primary infectious complex may become visible on a chest radiograph. Massive lymphohematogenous dissemination leading to meningitis, miliary, or disseminated disease occurs in 0.5–2% of infected children, usually no later than 2–6 months after infection. Clinically significant lymph node or endobronchial TB usually appears within 3–9 months. Lesions of the bones and joints usually take at least a year to develop; renal lesions may be evident 5–25 years after infection. In general, intrathoracic complications of the primary infection occur within the first year. Reactivation of TB is rare in infants and young children and, among adolescents, it affects females twice as often as males for unknown factors. Finally, the age of the child at acquisition of TB infection has a significant effect on the occurrence of both primary and reactivated TB. 

However, if young children do not suffer early complications, their risk of developing reactivated TB later in life is low. Conversely, older children and adolescents rarely experience complications of the primary infection but have a much higher risk of developing reactivated pulmonary TB as an adolescent or adult. Although protective immunity to TB in children is incompletely understood, several key attributes have been identified. As evidenced by children with underlying immunodeficiency, immune control of mycobacteria is dependent upon cell-mediated immunity (*Mtb*-specific T lymphocytes, dendritic cells, Toll-like receptors, γ-interferon (IFNγ), Tumor Necrosis Factor-α (TNFα), and interleukin), as well as macrophages and neutrophils [37]. Additionally, there is a distinctive risk profile of TB among children as younger children, and adolescents are at higher risk of progressing from infection to disease than children between 5 and 10 years old. As immune maturation proceeds, the risk for progressing to disease decreases.

HIV is a significant risk factor for the development of TB disease. There are limited data on incidence rates of TB in HIV-infected children, and rates vary significantly depending on the prevalence of HIV and TB in the community [38]. A study conducted in South Africa demonstrated that the incidence of TB in HIV-infected children was 42 times higher than in HIV-uninfected children [39]. 

#### 3.1.3. Mitochondrial changes in TB infection

Necrosis (involving cell swelling and lysis of plasma membranes) often coexists with apoptosis and both types of cell death are simultaneously observed in many infections. Although apoptosis of *Mtb*-infected macrophages is associated with diminution of the infection, preponderance of necrosis has been associated with increased bacterial growth [23,24,32]. There has been some indication that the condition of the mitochondria is the branch point leading either to necrosis or to apoptosis and Ca^2+^ acts as an intracellular messenger involved in cell death [23,24,32].

There are two main strains of *Mtb: Mtb* H37Ra (avirulent) or *Mtb* H37Rv (virulent), and infection of macrophages with H37Ra or H37Rv causes OMM permeabilization, characterized by CytC release [40]. Experiments with *Mtb* H37Rv suggest that these pathogens are able to override the detrimental effects of apoptosis by inducing necrosis, which results in uncontrolled mycobacterial growth. Mycobacteria have apoptotic as well as anti-apoptotic properties, and these seemingly contradictory findings have been attributed to the different virulence of *Mtb* strains [26]. The balance between proapototic and antiapoptotic proteins determines the flow rate of ions and water and, in consequence, the integrity of mitochondrial membranes [26,40].

An increase in intracellular Ca^2+^ protects mitochondria from irreversible *Mtb*-derived damage, promotes apoptosis, and inhibits alveolar macrophage necrosis and mycobacterial survival [41]. The effect of Ca^2+^ depletion on the mitochondria themselves is of critical importance leading to disruption of the Δψm and therefore the necrosis of the alveolar macrophage [41]. Transient dissipation of Δψm 6 h after infection is essential for the induction of macrophage necrosis by *Mtb*, a mechanism that allows further dissemination of the pathogen and development of the disease [42,43].

It is also important to note that the observed mitochondrial damage is a consequence of the NAD^+^ glycohydrolase activity of the TB necrotizing toxin (TNT) and does not require a direct interaction of TNT with the mitochondria, in contrast to other *Mtb* proteins associated with mitochondria [31]. TNT accounts for more than half of the loss of NAD^+^ in *Mtb*-infected macrophages. Low levels of cytosolic NAD^+^ directly decrease the concentration of NAD^+^ in the mitochondria, ultimately compromising ETC/MRC function and reducing the H^+^ gradient across the IMM. In this context, perturbations in the mitochondrial functioning of CII, CIII, and CIV among extrapulmonary gastrointestinal TB have been documented [44].

Of note, it is well known that mycobacterial infection of macrophages increases inducible nitric oxide synthase (iNOS) gene expression and, consequently, nitric oxide (NO) production. Remarkably, NO is also a potent inhibitor of cell respiration, by CIV inhibition. Blockade of respiratory chain CIV by NO, although related to mitochondrial dysfunction, may initiate a protective mitochondrial action by maintaining its Δψm, which results in prevention of apoptosis [26]. Some mycobacterial molecules involved in macrophage apoptosis have been identified [45] and are displayed, together with their main mitochondrial interactions (Table 2).

Importantly, *Mtb* infection may induce a quiescent energy phenotype in human monocyte-derived macrophages and decelerated flux through glycolysis and the TCA cycle. *Mtb* reduces mitochondrial dependency on glucose and increases mitochondrial dependency on fatty acids, shifting this dependency from endogenous fatty acids in uninfected cells to exogenous fatty acids in infected macrophages for survival under stress conditions. *Mtb* uniquely decelerates both glycolysis and OXPHOS to enter a state of metabolic quiescence and consequently decreases the rate of ATP production of the macrophage [48].

#### 3.1.4. TB Treatment in the Pediatric Population and Mitochondrial Involvement

Due to the common origin of mitochondria and bacteria, antibiotics are known to affect mitochondrial protein synthesis as an off-target consequence of their anti-bacterial function. In treating TB, it is of utmost importance to ensure adherence to medication to avoid potential re-infections and resistance in children. TB disease can be treated by the intake of several drugs for 6–9 months. There are 10 TB drugs currently approved by the US Food and Drug Administration (FDA). The first-line anti-TB agents isoniazid (INH), rifampin (RIF), ethambutol (EMB), and pyrazinamide (PZA) constitute the core of the treatment [49], and their associated mitochondrial dysfunction is shown (Table 3).

Rifapentine (RPT) is a bactericidal first-line treatment of TB but it has not been included due to the lack of data on its association with mitochondrial dysfunction [75,76,77,78,79]. Furthermore, it is not currently approved in Europe. In the United States, it is only used as treatment of latent infection. There are several clinical trials ongoing.

On the other hand, among second-line anti-TB drugs, not included in Table 3, both levofloxacin and moxifloxacin have been found to impair mitochondrial function. The former inhibits activities of mitochondrial ETC CI and CIII, leading to inhibition of mitochondrial respiration and reduction in ATP production [80,81]. Moxifloxacin promotes airway smooth muscle cell apoptosis by altering mitochondrial ΔΨm [82]. Other second-line anti-TB drugs are aminoglycosides, cycloserine, clofazimine, ethionamide, or the more recent bedaquiline and delamanid/pretomanid and the commonly used, linezolid, for which the off-target mitochondrial effects will be briefly discussed later in the text. 

Children who have been exposed to smear-positive adults with pulmonary TB but are free of symptoms, have negative immunodiagnostic TB tests (i.e., tuberculin skin test or interferon-gamma release assays -IGRA-) and a normal chest X ray are often put on INH primary chemoprophylaxis, if younger than 5 years of age or if they have other risk factors for the rapid development of the disease, such as being immunocompromised [36,83]. Failure to do so may result in rapid development of severe TB, during the incubation period. The child is treated for 8–12 weeks and the TST or IGRA is repeated; if the second test is positive, infection is documented and INH should be continued for 9 months, but if the second test is negative, TB infection is ruled out and the treatment can be stopped. All children with latent TB should also receive treatment to prevent the development of disease in the near or distant future [36]; the most common regimens are INH + RIF for 3 months, RIF for 4 months, or INH for 6–9 months. As a summary, children must receive anti-TB medication for several months. 

#### 3.1.5. Pediatric Studies of Mitochondrial Interaction in TB Infection

Difficulty in treating adults and children who have multidrug-resistant TB has led to increased interest in linezolid. Prolonged linezolid use is associated with high rates of hematologic and neurologic side effects based on inhibition of mitochondrial enzymes. In particular, the inhibition of mitochondrial protein synthesis diminishes respiratory chain enzyme content and thus limits aerobic energy production [84] without ultrastructural mitochondrial abnormalities and without mutations or depletion of mtDNA [85].

### 3.2. Enterobacteria 

The *Enterobacteriaceae* family comprises aerobic Gram-negative rods and facultative anaerobes. They are microbiologically characterized by not forming spores, fermenting glucose, not producing oxidase, and presenting variable mobility. They present a cytoplasmic membrane, a peptidoglycan coating and a complex cell wall that includes the capsule, which contains lipopolysaccharides and channels for the penetration of antibiotics and nutrients [86,87]. Enteric pathogens are a major source of morbidity and mortality throughout the world, including in children. It has been estimated that there are more than 3 million deaths associated with Gram-negative enteric pathogens worldwide due to diarrhea and enteric fever each year. The differences in disease manifestations are related to the different virulence factors present in the bacteria and the altered phenotypes that these virulence factors allow the organisms to employ during disease pathogenesis [88].

In this review, we are focusing on *Enterobacter cloacae*, *Proteus mirabilis*, *Escherichia coli*, and *Salmonella enterica* as their mitochondrial interactions in the pediatric population are widely studied and reported in the literature. 

First, *Enterobacter cloacae* can cause urinary tract and surgical wound infections, enteritis (especially in children), and even bacteremia. However, the most frequent presentations are nosocomial infections in immunocompromised patients [89], including children [90]. This pathogen presents a polysaccharide that potently inhibits cell proliferation of human osteosarcoma cells by inducing apoptosis through a loss of ΔΨm, release of CytC from the mitochondria into the cytosol, activation of caspase-9 and-3, cleavage of poly (ADP-ribose) polymerases (PARP), elevated ratio of Bax/Bcl-2 protein and overexpression of p53. As is known, the release of CytC from mitochondria into the cytosol is tightly regulated by Bcl-2 family proteins, which include pro-apoptotic Bax protein and anti-apoptotic Bcl-2 protein. Bax, known as a pro-apoptotic protein, is believed to have the ability to generate pores in the OMM, thus allowing the release of CytC into the cytoplasm to activate the pro-apoptotic caspase cascade. In contrast, Bcl-2, as one type of anti-apoptotic protein, inhibits CytC release, resulting in prevention of apoptosis initiation. These two apoptosis-modulating proteins are also regulated by the gene p53, the tumor suppressor gene, which functions as a cellular emergency response system to induce cell growth arrest or apoptosis via the equilibrium between Bax and Bcl-2. Polysaccharide from *E. cloacae* induces apoptosis in osteosarcoma cells via alteration of Bax, Bcl-2, and p53 protein expression [91]. 

Second, *Proteus mirabilis* is a common cause of urinary tract infection in childhood, after *E. Coli* [92]. This pathogen has been related to pathological changes in the mitochondria, in particular, intra-mitochondrial crystals. This is due to the large stores of calcium in the mitochondria and the endoplasmic reticulum. When the pH level increases greater than 7.3 and the solubility of calcium phosphate is exceeded, calcium precipitation occurs. Activity and mechanical damage by crystals eventually leads to nuclear destruction and disruption of calcium levels. In infected cells, mitochondrial swelling, inflammation, and destruction is visible, suggesting apoptosis involving ROS, CytC, caspase-9, and caspase-3 [93]. 

Third, *Escherichia coli* is a main causative agent of urinary tract infections worldwide and of childhood diarrhea in developing countries. The EspF effector protein is the product of the espF gene found at the enterocyte effacement site, the key pathogenicity island carried by enteropathogenic *E. coli* and enterohemorrhagic *E. coli.* Importantly, EspF, whose N-terminus is a mitochondrial targeting signal, plays a role in the permeabilization of the mitochondrial membrane induced by enteropathogenic *E. coli* infection. Furthermore, EspF is associated with the release of CytC from the mitochondria to cytoplasm and cleavage of caspase-9 and caspase-3. These findings indicate a role for EspF in initiating the mitochondrial death pathway, thus suggesting that intracellular EspF is targeted to mitochondria. Mitochondrial proteins often reside in an N-terminal mitochondrial targeting signal that can be cleaved by a specific peptidase after import into the mitochondrial matrix [94].

Fourth, *Salmonella enterica* is one of the top four causes of diarrheal disease. Most cases of salmonellosis are mild, although they can sometimes be fatal. Every year 550 million people get sick, of whom 220 million are children under the age of 5 [95]. There are a few studies mentioning mitochondrial changes in children with salmonellosis, although interestingly and related to therapeutic approaches, mitochondrial components of monocytes from young children with salmonellosis (6 months to 2 years), have been assessed in order to test the effectiveness of low intensity lasers on functional cell status. These have reported normalization of mitochondrial components and improvement of clinical symptoms [96].

#### Pediatric Studies of Mitochondrial Interaction in Enterobacteria

From the Enterobacteriaceae family, *E. cloacae*, *P. mirabilis, E. Coli*, or *S. enterica* have the same mitochondrial influence, as the pathogens are associated with loss of ΔΨm, release of CytC, and activation of caspase-3 and -9 [92,94,95]. Most of the aforementioned alterations are due to the precipitation of intra-mitochondrial calcium and the increase in ROS, as reported in children [93].

### 3.3. Staphylococcus aureus 

*Staphylococcus* is a group of bacteria that includes 30 types, from which *Staphylococcus aureus* (*S. aureus*) is the principal cause of staph infections. *S. aureus* is a Gram-positive member of the *Firmicutes* phylum of the *Micrococcaceae* family within the Bacillus class [97]. Humans are a natural reservoir of *S. aureus*. Between 30% and 50% of healthy adults are colonized, and between 10% and 20% permanently colonized [97,98]. *S. aureus* is a commensal organism and also an important opportunistic human pathogen, causing a variety of community and hospital-associated pathologies, including bacteremia-sepsis, endocarditis, pneumonia, osteomyelitis, arthritis, and skin diseases [97,99]. Under normal conditions, *S. aureus* does not cause infections, this only occurs in immunocompromised patients in whom the persistence of the bacteria in the host leads to disease risks [100]. Pathogenic infections are generally initiated by penetration of skin or mucosal barriers, allowing bacteria to access adjacent tissues or the bloodstream [101]. The release of toxins derived from *S. aureus* into the skin and other organs can cause various types of skin rashes and general symptoms, as in the case of toxic shock syndrome or acute diarrheal disease.

The urgency of *S. aureus* over the past decade in many settings has been facilitated not only by mechanisms of resistance to bacterial antibiotics but also by the emergence of new clonal types of *S. aureus* with increased expression of virulence factors and their ability to neutralize the host’s immune response [97,100]. Prevention of the spread of *S. aureus* infection is based on the use of adequate contact precautions and infection control procedures that have, so far, not been fully effective [101]. 

#### 3.3.1. Structure and Replication Cycle

The genus Staphylococcus is formed by Gram-positive cocci, with a diameter of 0.5–1.5 μm, grouped as single cells, in pairs, tetrads, short chains, or forming bunches of grapes. They are non-mobile bacteria, not sporulated, mostly lacking capsule, although there are some strains that develop a slime capsule, and are facultative anaerobes. Most staphylococci produce catalase, the enzyme capable of splitting hydrogen peroxide into water and free oxygen; a main feature used to differentiate the *Staphylococcus* genus from the *Streptococcus* and *Enterococcus* genera, which are catalase negative [100]. The cell wall of staphylococci has teichoic acids attached, which do not exist in the micrococci and, finally, another difference is the composition of the cytochrome and menaquinone of the respiratory chain present in staphylococci [100].

*S. aureus* is widely distributed among primates but is not restricted to them exclusively, e.g., it produces mastitis in cattle and sheep. In humans, the nasal location of *S. aureus* allows its spread and, as a consequence, the spread of its multi-resistance to antibiotics such as methicillin (MRSA) [100].

The complex physiology of *S. aureus* makes the organism highly versatile, adaptable, and capable of resisting many host defense mechanisms [99]. The pathogenicity of infections by *S. aureus* is related to various components of the bacterial surface; in general, peptidoglycans and teichoic acids, in addition to protein A. Thus, the pathogenesis caused by this microorganism arises when there exists a combination of these virulence factors with decreased defenses of the host [102]. These conditions favor *S. aureus*, allowing it to present important virulence characteristics resulting in particular damage.

Patients with *S. aureus* infections often become infected with the same strain that colonizes their nostrils; colonization also allows transmission between individuals in the hospital and in the community. For an adequate survival and invasion of the host, this whole system of virulence factors must form part of a cell–cell communication system, mediated by proteins produced by the bacteria and depending on environmental factors. This system can activate a large number of genes coding for virulence factors. *S. aureus* has proteins on its surface known for inhibiting phagocytosis and opsonization by the human complement system. The recognition by complement and immunoglobulins of receptors are blocked by protein A, on the cell wall, which binds to a certain portion of the IgG immunoglobulin. *S. aureus* also produces molecules that can inhibit neutrophil recruitment, phagocytosis, and bacterial recognition [100]. Perhaps one of the most salient features of *S. aureus* is its ability to produce a variety of toxins that are targeted at human blood cells. These toxins include hemolysin-α, hemolysin-β and hemolysin-γ, Panton–Valentine leukocidin, and phenol-soluble β-modulin, which have been recently discovered [100,103].

#### 3.3.2. *Staphylococcus aureus* in the Pediatric Population

Even though *S. aureus* is known to be a common cause of bacteremia in children leading to significant morbidity and mortality, there are quite few studies of *S. aureus* in pediatrics. Little is known about the frequency of nasal colonization by MRSA in young children, but in some cohorts 17.5–18.1% of the children were colonized with *S. aureus* and 1.3% with MRSA, and the bacteremia case-fatality ratio has been described as high as 14.1%, in some developing populations [104,105]. MRSA infection has recently become a difficult problem worldwide, which requires special attention in pediatrics. Strain characteristics of pediatric MRSA-infected children have been assessed through whole genome sequencing, describing a particular tropism of a specific clone in pediatric patients and while susceptible to fluoroquinolones and tetracyclines, these should be prescribed in children with caution [106].

#### 3.3.3. Mitochondrial Changes in *S. aureus* Infection

*S. aureus* promotes mitochondrial alterations by a panoply of interactions with the organelle, as described below. *S. aureus* infection activates mitochondrial changes when, subsequently to phagocytosis of the bacterium, caspase-8 is activated followed by the progressive interruption of Δψm [107], ultimately leading to the production of ROS. Mitochondrial ROS generation has been shown to drive bactericidal macrophage activity against MRSA [108]. Of note, the reversible caspase-8 inhibitor, IETD-FMK, prevents disruption of Δψm and prevents CytC release from monocytes exposed to *S. aureus* [109].

Another example of mitochondrial interactions occurs during acute infection of lung lesions, in which *S. aureus* activates a ubiquitin E3 ligase component, the so-called Fbxo15, which mediates proteasomal degradation in epithelia, resulting in decreased availability of cardiolipin, the main lipid in the IMM, and altered mitochondrial function [110]. Importantly, *S. aureus* secretes a pore-forming toxin, Panton–Valentine leucocidin, that has recently been associated with necrotizing pneumonia. Panton–Valentine leucocidin-induced apoptosis has been associated with rapid disruption of mitochondrial homeostasis and activation of caspase-9 and caspase-3, suggesting that this induced apoptosis is preferentially mediated by the mitochondrial pathway [111,112,113]. Finally, MRSA actively prevents the recruitment of mitochondria to the area around the vacuoles in which the bacteria reside to prevent intracellular death, prompted by caspase-11, allowing for the survival of MRSA within macrophages [114]. 

#### 3.3.4. *S. aureus* Treatment in the Pediatric Population and Mitochondrial Involvement

Invasive *S. aureus* infections are a leading cause of morbidity and mortality in both hospital and community settings, especially with the widespread emergence of virulent and resistant *S. aureus* strains [115,116]. The clinical use of methicillin has led to the appearance of MRSA [117]. However, there are other antibiotics available to treat this infection [118]. Again, taking into account the common origin of mitochondria and bacteria, antibiotics are known to affect mitochondrial protein synthesis. Anti-*S. Aureus* drugs along with the mitochondrial damage related to their administration are depicted below (Table 4).

Of note, the bactericides oxacillin, cloxacillin, dicloxacillin, and nafcillin, belonging to the beta-lactam family, which prevent the formation of the bacterial cell wall [109,110] as well as the bacteriostatic clindamycin, belonging to the lincosamide family, have not been considered in the table due to the lack of pediatric studies (neither has clindamycin been associated with mitochondria in the literature). However, it should be mentioned that the former promote mitochondrial oxidative impairment through ROS overproduction [138] via the disruption of the TCA cycle and ETC [139], as well as decrease Δψm [139]. Daptomycin is another antibiotic from the lipopeptic family, which has been demonstrated to present high clinical success rates against a wide variety of infections and is well tolerated in children and adolescents [140]. It has been excluded from Table 4 due to the lack of information on its mitochondrial impact.

In general, antibiotics in the beta-lactam family are the first choice for the treatment of methicillin-sensitive infections. Vancomycin has been used for decades for the antibiotic treatment of methicillin resistance [101].

#### 3.3.5. Pediatric Studies of Mitochondrial Interactions in *S. aureus* Infection

High risk groups for *S. aureus* infection include not only patients undergoing surgical procedures and individuals undergoing immunosuppressive or cancer therapy but also infants with low birth weight and young children. Occasionally, it has been observed that people who have *S. aureus* infection have Panton-Valentine leukocidin localized to the mitochondrial membrane, inducing mitochondrial apoptosis [116]. Bacterial sepsis induces mitochondrial injury resulting in depressed metabolism, while biogenesis restores mitochondrial content and function [141].

### 3.4. Meningitis

Meningitis is an infection characterized by inflammation of the meninges that in 80% of cases is caused by viruses, in 15–20% of cases, it is caused by bacteria and in the rest of cases, it is due to poisoning, fungi, medications, and other diseases. Here, we classify meningitis according to the causative agent [142] and relevance in the pediatric population [143] (Table 5). 

The microorganisms that cause 95% of the cases of bacterial meningitis, i.e., *Neisseria meningitidis (N. meningitidis*, meningococcus) and *Streptococcus pneumoniae (S. pneumoniae, pneumococcus*), are habitual residents of the nasopharynx and oropharynx sites, where they do not normally cause damage. However, for unknown reasons, they can eventually pass into the blood reaching and colonizing the meninges [144]. Herein, we review the potential mitochondrial interactions of the two most relevant pathogens for meningitis in pediatrics, *N. meningitidis* and *S. pneumoniae*.

*N. meningitidis* is a Gram-negative anaerobic pathogen belonging to the β subgroup of proteobacteria that colonizes the nasopharynx in up to 35% of healthy people. They only colonize human hosts with no other known reservoirs [145]. It infects 500,000 to 1.2 million people and kills between 50,000 and 135,000 per year [146,147,148] causing significant morbidity and mortality in children and young adults worldwide through epidemic or sporadic meningitis and/or sepsis [146]. On the contrary, *S. pneumoniae* is a spherical, facultative anaerobic member of the genus Streptococcus, which is part of the normal upper respiratory tract flora and the second most common cause of meningitis in children older than 2 years [149]. Both pathogens are usually found in pairs (diplococci) [150,151].

Direct transmission of both meningococci and pneumococci occurs by sharing respiratory and throat secretions (saliva or spit) [145,152] and, in exceptional cases, can be a cause of neonatal infections. The most frequent symptoms associated with meningitis by both pathogens are headache, neck stiffness, fever, photophobia or phonophobia, and altered consciousness [153]. Often, particularly in young children, only nonspecific symptoms such as irritability and drowsiness occur. The existence of a skin rash is common in meningococcemia [154]. Bulging of the anterior fontanel can also occur in infants [155]. 

Infections due to *N. meningitidis* can cause sepsis and meningitis once it reaches the bloodstream and nervous system, respectively, [156] and present as a spectrum of clinical disease, with meningitis and septicemia being the most common, but it also includes pneumonia, septic arthritis, pericarditis, conjunctivitis, and urethritis [148]. Infections due to *S. pneumoniae* mainly cause pneumonia and otitis media, but also other diseases and symptoms including pneumococcal meningitis, fever and chills, cough, rapid breathing, difficulty breathing, chest pain, and sepsis [157].

There are 13 serogroups of *N. meningitidis* based on different capsular polysaccharide structures, but only six of them (A, B, C, W-135, X, and Y) cause the majority of life-threatening disease. During some epidemics, the incidence increases in older children and adults [147]. In endemic situations, serogroup B is more common in infants, serogroup C in adolescents, and serogroups B or Y in older adults, although this depends on the geographical zone. Serogroup A carriage has been observed as the most prevalent in older children and young adults in African cohorts [147]. All age groups are at risk of invasive meningococcal disease, but infants and adolescents are particularly vulnerable due to the disappearance of maternal antibodies early in life and the high rate of nasopharyngeal colonization [155].

There are more than 90 known serotypes of *S. pneumoniae*, but only 12 (1, 3, 4, 5, 6, 7, 8, 9,14, 18, 19, and 23) present the highest clinical impact, responsible for >80% of invasive pneumococcal infections [158]. Specifically, serotypes: 6, 9, 14, 18, and 23 [159] are the ones that most often cause meningitis. Resistance to antibiotics has been mainly associated with serotypes 6, 14, 19, and 23, and these are the most frequently isolated serotypes in children under 2 years of age are associated with prolonged carrier states, and are easily reacquired [160]. 

Meningitis progresses very rapidly, so early diagnosis and early treatment are important to prevent serious sequelae and death [161]. Death occurs in 6–10% of cases and sequelae in 4.3–11.2% of cases.

#### 3.4.1. Structure and Replication Cycle

The infection may be acquired when these micro-organisms residing in the normal nasopharynx and oropharynx habitat end up colonizing the meninges through the blood, through nearby injury (after fractures, fissures, and lumbar punctures, among others) or through contiguous spread from a nearby suppurative focus [144].

The expression of the capsule polysaccharide plays a key role in meningococcal pathogenesis [148]. The virulence of *N. meningitidis* is influenced by multiple factors, such as capsule polysaccharide expression, surface adhesive protein expression (outer membrane proteins including pili, PorA and B porins, Opa and Opc adhesion molecules), sequestration mechanisms and endotoxin production (lipooligosaccharide, LOS) [146]. Once meningococci penetrate the mucosal barrier of the upper respiratory tract and adhere to human epithelial cells, a series of interactions take place, resulting in an effect on the epithelial surface and the formation of microcolonies. Viable meningococci can be phagocytosed by respiratory or non-ciliated epithelial cells or escape, thus reaching the submucosa or directly invading damaged epithelial surfaces [147].

The pathogenicity of *S. pneumoniae* is characterized by its capsule, which is essential due to its ability to block opsonization through the complement system, and phagocytosis by cells of the immune system; along with the wall, made up of a network of peptidoglycan chains, lipids, and teichoic acids. They play an important role in the processes of colonization, adherence, inflammation, and bacterial invasion [162,163].

Once in the bloodstream, bacteria enter the subarachnoid space at places where the blood–brain barrier is vulnerable, such as the choroid plexus. The large-scale inflammation that occurs in the subarachnoid space during meningitis is not a direct result of bacterial infection but can largely be attributed to the response of the immune system to the entry of bacteria into the central nervous system. When the components of the bacterial cell membrane are identified by the cells of the brain’s immune system (astrocytes and microglia), they respond with the release of large amounts of cytokines. The blood–brain barrier presents changes, leading to “vasogenic” cerebral edema (swelling of the brain due to leakage of fluid from the blood vessels). Large numbers of leukocytes entering the cerebrospinal fluid cause inflammation of the meninges and lead to interstitial edema (edema due to intercellular fluid). In addition, the walls of the blood vessels themselves become swollen (cerebral vasculitis), leading to decreased blood flow and a third type of edema, the so-called cytotoxic edema [159].

#### 3.4.2. Meningitis in the Pediatric Population

In regards of meningococcal disease, the highest incidence of the disease occurs in childhood and the most susceptible children are from 6 to 24 months, in whom passively transferred maternal antibodies have already disappeared [164]. Despite the advances in the treatment of this disease, mortality in the United States is as high as 8% with neurological sequelae observed in up to 31% of cases [165]. 

#### 3.4.3. Mitochondrial Changes Derived from Meningitis Pathogens 

In regards of *N. meningitidis*, the virulence factor NHBA binds heparin through a conserved region rich in arginine that is the target of two proteases, the meningococcal NalP and human lactoferrin, responsible for the binding of heparin and heparan sulfate. Binding to heparin improves the survival of the bacterium in human serum, while binding to heparan sulfate has often been linked to the ability to bind and invade host cells; heparan sulfate is produced as a proteoglycan on the surface of many types of cells [155,156].

The NHBA can be cleaved by NalP, generating a fragment called C2 that maintains an arginine-rich domain or on the other hand, it can be cleaved by human lactoferrin, generating the fragment called C1, in which the arginine-rich domain is absent. Both fragments are released from the bacteria into the surrounding environment. Since the C2 fragment retains the domain responsible for binding to heparin, it is plausible that it exercises its own biological role. The C2 glue accumulates rapidly in the mitochondria where it induces the production of ROS. This is necessary for phosphorylation of the binding protein and for its internalization, which, in turn, is responsible for endothelial leakage/permeability [156]. Therefore, the integrity of the endothelial barrier can be disturbed by ROS, which worsens the pathological conditions in the presence of the bacteria.

On the other hand, *S. pneumoniae* is the most common and aggressive cause of bacterial meningitis and induces a novel AIF–dependent form of brain cell apoptosis. Specifically, two pneumococcal toxins, pneumolysin and H_2_O_2_, produce mitochondrial damage and apoptosis. Both toxins induce an increase in intracellular Ca^2+^ and trigger the release of AIF from mitochondria [166,167].

#### 3.4.4. Meningitis Treatment in the Pediatric Population and Mitochondrial Involvement 

The treatment of meningitis in the pediatric population depends on the causative agent. For instance, the inclusion of conjugated vaccines against *H. influenzae* type b; meningococcal serogroup B, C, ACWY; and pneumococcus in systematic vaccination schedules has caused a significant decrease in the incidence of these diseases. For bacterial meningitis, different antibiotics, with subsequent mitochondrial associated changes, are available for treatment (Table 6). As previously depicted, due to the common origin of mitochondria and bacteria, antibiotics are known to affect mitochondrial protein synthesis as an off-target consequence of their anti-bacterial function. Specific mitochondrial interactions derived from these antibiotics are provided (Table 6).

In epidemic conditions in areas of Africa with limited resources or poor health infrastructure, the drug of choice is ceftriaxone [150,178,179]. In children with bacterial meningitis caused by pneumococci or *H. influenzae* type B, adjuvant treatment with dexamethasone significantly reduces the risk of sensorineural hearing loss and ataxia [178].

In regards of *N. meningitidis*, there are three types of vaccine, some of which have been available for more than 30 years. Vaccines can be monovalent (group C), bivalent (groups A and C), trivalent (groups A, C, and W), or tetravalent (groups A, C, Y, and W135). The first vaccine against group B has been recently developed and combines 4 protein components [148,179]. This vaccine, named “Bexsero” and “Trumemba,” specifically targets a surface-exposed lipoprotein ubiquitously expressed by *N. meningitidis* strains, the so-called Neisseria heparin-binding antigen (NHBA).

In the case of *S. pneumoniae*, several vaccines have been developed. The WHO recommends routine childhood pneumococcal vaccination. Thus, it is incorporated into the childhood immunization schedule, along with the meningococcal conjugate vaccine, in a number of countries [180]. 

#### 3.4.5. Pediatric Studies of Mitochondrial Interaction in Meningitis Infection

In meningitis, brain cells produce cytokines, chemokines, and other pro-inflammatory molecules in response to bacterial stimuli, and polymorphonuclear leukocytes are attracted and activated and release large amounts of O^2−^ and NO, leading to ONOO^−^ formation generating oxidative stress. This cascade leads to lipid peroxidation, mitochondrial damage, and breakdown of the blood–brain barrier, thus contributing to cell injury during neonatal meningitis [181]. Nitric oxide, which is a specific inhibitor of CIV MRC, is very likely to play a role in the physiopathological mechanisms of bacterial meningitis in children. As shown in in vitro studies, NO is toxic to endothelial cells, as well as to neurons and, thus, may be responsible for neurological sequelae in bacterial meningitis. Increased levels of NO can also inhibit mitochondrial respiration, enhancing anaerobic glycolysis [182].

## 4. Parasitic Infectious Processes and Mitochondrial Involvement

### 4.1. Malaria

Malaria is one of the three major global infectious health threats [183] causing approximately 2.7 million deaths per year, mainly among young children under the age of 5 [184]. The disease is caused by *Plasmodium* parasites, and mosquitoes are essential for the spread of the disease [185]. There are different malarial parasites, including *Plasmodium falciparum, Plasmodium malariae, Plasmodium brasilianum, Plasmodium inui, Plasmodium vivax, Plasmodium ovale, Plasmodium cynomolgi, and Plasmodium bergei* [186]. Of these species, *P vivax* and *P falciparum* cause 95% of infections [187,188], although the latter is the main cause of malaria in humans [188].

Symptoms include fever, headaches, and vomiting, and they appear 10–15 days after the bite from a mosquito carrying the parasite. In general, malaria causes hemolysis and alters the contribution of blood to vital organs, putting the patient’s life in danger [189].

Severe or complicated malaria occurs in most cases by *P. falciparum* and *P. knowlesi*. It presents with an alteration of consciousness, delirium, seizures or coma (cerebral malaria), extreme prostration, inability to feed, respiratory distress, circulatory collapse, jaundice, muco-cutaneous hemorrhages (petechiae), and acute kidney failure (rare in children). Regarding hematological alterations, there is severe anemia (Hb < 5 gr%) due to acute intravascular hemolysis (sometimes triggering hemoglobinuria) and hypersplenism (common in children and predominantly those under 2 years), secondary hyperbilirubinemia, thrombopenia, and disseminated intravascular coagulation. Lactic acidosis and severe hypoglycemia (blood glucose < 40 mg/dL) also occur due to increased consumption of glucose by the parasite and liver disease. Children with severe malaria should be hospitalized, since it is fatal in most cases without specific treatment and in spite of treatment still reaches mortality rates of 10–20% [190].

#### 4.1.1. Structure and Replication Cycle

**Figure 5:**
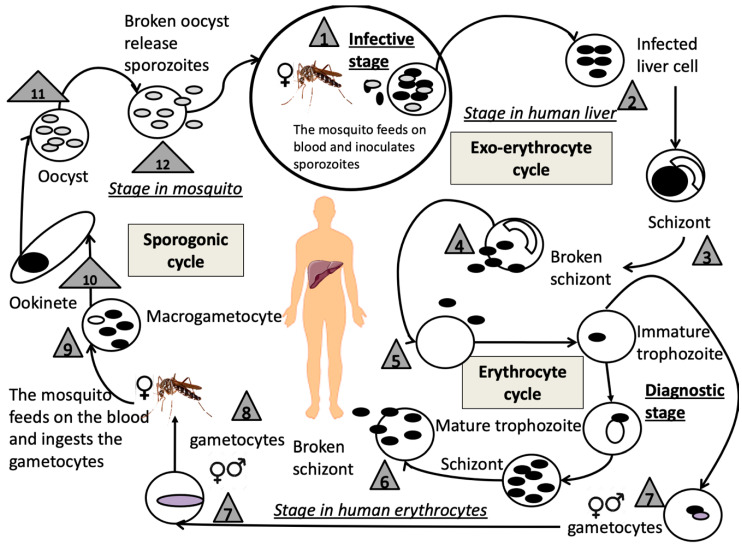
Replication stages in malaria infection. The sporozoites enter the circulation after the mosquito’s bite. They are then transported through the blood to hepatocytes in the liver, initiating the exo-red cell cycle. There, they rapidly multiply within hepatocytes by multiple cycles of asexual division and transform into merozoites that enter the bloodstream and leave the liver. Merozoites invade red blood cells, initiating the erythrocytic cycle. As the nucleus begins to divide, the trophozoite is now called a developing schizont. The mature schizont contains merozoites that are released into the bloodstream. Although many merozoites are destroyed by the immune system, others immediately invade red blood cells, in which a new cycle of erythrocytic schizogony begins. After several generations of erythrocytes, male and female gametocytes develop from some merozoites (sexual cycle). With the union of the gametes, the egg is generated in the mosquito’s intestine. The egg is mobile and will give rise to an oocyst that will divide again and give sporozoites.

Every patient infected with malaria (regardless of whether or not they show symptoms) has the parasite go through the exact same life-cycle, morphological changes, and human–parasite interactions [186]. From the time of the mosquito bite to approximately 1 week later, the patient remains asymptomatic [188]. The replication cycle is highly complex and has been represented to facilitate understanding (Figure 5). Specifically, the vector is the female mosquito, of the *Anopheles* genus and the *Culicidae* family, which carries *Plasmodium* in its salivary glands so that after the mosquito’s bite, the sporozoites enter the circulation. In approximately 60 min, the sporozoites are transported through the blood to hepatocytes in the liver, thereby initiating the exo-red cell cycle. There, they rapidly multiply within hepatocytes by multiple asexual division and transform into merozoites that enter the bloodstream. Once these merozoites leave the liver, they invade the red blood cells, thus initiating the erythrocytic cycle. As the nucleus begins to divide, the trophozoite is now called a developing schizont. The mature schizont contains merozoites that are released into the bloodstream. Although many merozoites are destroyed by the immune system, others immediately invade red blood cells, in which a new cycle of erythrocytic schizogony begins [187].

After several generations of erythrocytes, male and female gametocytes are developed from some merozoites. When an uninfected female Anopheles bites a patient and acquires gametocytes, the Plasmodium sexual cycle begins. With the union of the gametes, the egg is generated in the mosquito’s intestine. The egg is mobile and will give rise to an oocyst that will divide again and give sporozoites ready to infect again, upon reaching the mosquito’s salivary glands [187].

Plasmodium parasites are eukaryotes and therefore contain mitochondria, since they live in aerobic hosts [185]. Most parasites do not use the oxygen available within the host to generate ATP, but instead use anaerobic metabolic pathway systems [191]. Furthermore, all parasites have their own life cycle and, in many cases, use aerobic metabolism during “their free life stage” outside the host. Plasmodium depends mainly on monocytosolic glycolysis but not on OXPHOS, although this is essential for its survival. For this reason, there is very little complete oxidation of glucose nor a subsequent increase in by-products such as lactate and pyruvate [192]. The mitochondria of the malaria parasite *Plasmodium falciparum* are morphologically different between the asexual and sexual blood stages (gametocytes). However, Plasmodium has all the genes necessary for TCA cycle and the existence of CI, CII, CII, and CIV suggests that the biochemically active ETC operates in this parasite. There is also an alternative branching pathway for electron transport, which includes an anaerobic function of CII [188,193]. Some of the functions of the mitochondria in the parasite include the coordination of pyrimidine biosynthesis, the ETC, and the utilization of oxygen through dihydroorotate dehydrogenase and CoQ [188]. 

#### 4.1.2. Malaria in the Pediatric Population

Every 2 min, a child dies from malaria and every year more than 200 million new cases are estimated to occur in children [194]. Although some countries have drastically reduced the number of cases and deaths since 2000, in recent years, there has been a stagnation in reduction and, worryingly, malaria is progressing in some countries. Children under the age of 5 are the most vulnerable group; in 2018, they accounted for 67% (272,000) of all malaria deaths worldwide [194]. Furthermore, malaria is one of the most severe and life-threatening infections affecting international pediatric travelers, and it is more frequent in child travelers who return to their countries of origin (often less developed than their country of residence) for the purpose of visiting friends and relatives. Children can develop severe parasitemias rapidly and are at increased risk for associated complications, such as seizures, shock, coma, and death, with the added problem that they may initially present with nonspecific symptoms that often lead to delayed diagnosis [195].

#### 4.1.3. Mitochondrial Changes in Malaria Infection

Despite the fact that evolutionary pressure from *Plasmodium falciparum* malaria has favored a large number of human gene adaptations, there are surprisingly a few investigations on the effects of malaria on human mitochondrial sequence variation. *Plasmodium falciparum* infection can cause severe malaria anemia with insufficient tissue oxygenation, lactic acidosis, and death [196].

Asymptomatic children can temporarily maintain their status by orchestrating active gene regulation through chromatin remodeling [197]. This likely affects the production of immunoglobulin chain transcripts found repressed in asymptomatic children, but specifically activated in uncomplicated pediatric malaria. On the contrary, in symptomatic malaria, basophil and eosinophil transcripts remain suppressed. More specifically, interferon-alpha inducible protein 27 (IFI27) levels have been found to correlate with clinical parameters and thus could represent a potential indicator of parasitemia, along with hemoglobin and lactate levels. The latter occurs because persistent oxygen deficiency leads to a replacement of intracellular aerobic respiration with anaerobic glycolysis and excessive production of lactic acid [197]. IFI27 is associated with, or inserted into, the mitochondrial membrane and its transient expression leads to a decrease in the number of viable cells and an increased sensitivity to apoptosis induced by DNA damage. This suggests the involvement of IFI27 in the mechanisms of apoptosis that generate lymphopenia during severe anemia due to malaria.

Approximately 50% of Ugandan children with severe malarial anemia present elevated blood lactate levels [196]. In this study, mitochondrial gene sequences among a cohort of children with or without lactic acidosis in the context of severe malaria anemia were investigated [196]. It has been suggested that the determinants of high blood lactate levels in severe malarial anemia patients may be related to genetic polymorphisms outside the mitochondrial genome, or to other factors. Alternatively, variation in other nuclear genes such as those in the glycolytic pathway, Krebs cycle, or those that affect NAD^+^/NADH levels may influence blood lactate levels during severe anemia. In addition, severe malarial anemia lactic acidosis may also be related to factors unrelated to genetics, such as microvascular physiology, nutritional status, or duration of disease. Increased blood lactate, an important marker for decreased survival in severe malarial anemia, despite being a consequence of mitochondrial impairment, does not appear to be strongly associated with mitochondrial DNA sequence variation, but could be related to variations in those nuclear genes influencing mitochondrial function [196]. 

#### 4.1.4. Malaria Treatment in the Pediatric Population and Mitochondrial Involvement

Chemoprophylaxis depends on the species of the parasite, area of origin (and Plasmodium resistance rates in that country), and clinical situation of the child (severity criteria) [190]. The parasite’s mitochondria has been detected as a main potential target for antimalarial drugs and, in particular, the mitochondrial ETC, as it has a relatively limited function while the parasite is developing [188] (Table 7). Of note, it is conceivable that if the treatment targets the mitochondria of the parasites, it may cause off-target effects in the mitochondrial function of the patients.

Depending on the parasite involved and the clinical situation at diagnosis, the treatment can be administered on an outpatient basis or intravenously. 

If malaria is diagnosed early, it can usually be cured within about 2 weeks. However, many people living in areas where malaria is endemic are repeatedly infected and never completely recover between consecutive bouts of this disease. Without treatment, the disease can be fatal, especially in malnourished children [207].

Finally, there is a vaccine, known as Mosquirix, which is an injectable option that provides partial protection against malaria in young children and that is being evaluated in sub-Saharan Africa as an instrument of complementary control that could be added to other preventive, diagnostic, and therapeutic measures recommended by the WHO [190].

## 5. Discussion

To our knowledge, this is the first time that such mitochondrial molecular events, both at the genetic and at the functional level, associated with the main bacterial infections and their treatments, are reviewed in the pediatric population. In fact, a major observation of the present review, is the lack of information relating to the role of mitochondria in most bacterial infective processes in the pediatric population.

Both intracellular and extracellular bacteria have been shown to modify host metabolism by disturbing mitochondrial homeostasis and function. Recently, several intracellular bacterial pathogens have been shown to modulate mitochondrial functions to maintain their replicative niche [208]. Thus, infection induces mitochondrial changes in infected macrophages, triggering modifications of the host metabolism that lead to important immunological reprogramming.

Importantly, mitochondrial interactions and toxicity are ultimately determined by bacterial load as well as by the drug selected to treat such infections. These interactions eventually and frequently turn out to be reversible once the pathogen is eradicated or the therapeutic agent is interrupted.

A main mitochondrial hallmark related to most infective processes is the increase in mitochondrial ROS that occurs in many bacterial (e.g., enterobacteria) and parasitic (e.g., malaria) infections. For example, during meningitis, the integrity of the endothelial barrier can be disturbed by ROS, which worsens pathological conditions in the presence of the bacteria [156]. 

Interestingly, ROS not only are a by-product of oxidative respiration but also regulate signaling pathways such as signal transducers and activators of transcription, and phosphoinositide 3-kinase pathways. Hence, an increase in ROS as a cellular stress signal has to be avoided or counteracted during the establishment of a persistent infection [209]. ROS generation and subsequent oxidative stress often lead to apoptosis. Mitochondrially driven apoptosis caused by bacterial infections has also been documented. Such is the case of the Enterobacteriaceae family, and others such as *S. aureus*, which also cause mitochondrial impairment. Interestingly, all these infections are characterized by promoting loss of ΔΨm, release of CytC, activation of caspase-3 and -9 [94], ultimately leading to mitochondrial driven cell death [31,190]. In addition, worth noting is the increase in Bax/Bcl-2 proteins in *E. clocae* and the overexpression of p53 [91] in *P. mirabilis*, also associated with intramitochondrial calcium precipitation and ROS increase [93], the latter also observed in *S. aureus* [26,210]. 

However, mitochondrial damage derived from bacterial infections goes beyond ROS generation and apoptosis. First, many infectious processes caused by different pathological agents not only are related to ROS overproduction but also share some other similar molecular events, such as inflammatory mechanisms [211]. Second, it is known that disruption of mitochondrial integrity has been identified as a key virulence strategy of bacterial pathogens [212]. Third, most products derived from bacterial infection, such as nitric oxide, are widely known to be specific inhibitors of complex IV of the mitochondrial respiratory chain [213]. Lastly, metabolic switching from an aerobic to anaerobic state and vice versa, has been documented during bacterial infections [208]. Mitochondrial performance is highly adaptive during an infectious process of either bacterial or parasitic origin. In malaria, for example, oxygen deficiency leads to a replacement of intracellular aerobic respiration with anaerobic glycolysis and excessive production of lactic acid [197]. Interestingly, sometimes the mitochondrial and cellular changes triggered by the infective process are destined to protect the cell. In TB, the increase in intracellular Ca^2+^ protects the mitochondria from irreversible damage caused by the pathogen, *Mtb*, and inhibits macrophage necrosis. Of course, deleterious effects leading to mitochondrial damage are also present in TB, mainly related to the H37Rv*Mtb* strain, by exerting changes in the Δψm, release of CytC, and modification of mitochondrial dynamics [26,31]. 

As observed in this review, not only the pathogens but also their treatments are frequently associated with mitochondrial changes. This is explained, as already mentioned, by the shared bacterial-mitochondrial origin [6]. Accordingly, antibiotics have been reported to inhibit mitochondrial protein synthesis, due to the common origin of mitochondria and bacteria, described by Margulis in the endosymbiont hypothesis [214]. This is the case of linezolid-derived inhibition of cytochrome-c-subunit protein of complex IV, as reported by our group [123].

In clinical practice, it is occasionally difficult to differentiate whether mitochondrial abnormalities are exclusively related to the infection itself or to its antimicrobial treatment [3] but, importantly, molecular events have been correlated with the onset of clinical symptoms in the pediatric population, meaning mitochondrial alterations are more evident in children presenting with clinical manifestations than in those without [215]. The vulnerability of pediatric patients highlights the importance of longitudinal studies assessing mitochondrial changes and their derived clinical consequences over time. 

In other cases, mitochondria of the pathogen itself turns out to be the main therapeutic target to treat the infection and pharmacological inhibition of a given mitochondrial function may represent a key step to avoid pathogen replication. Such is the case with pharmacological inhibition of complex III, a well-defined drug target for the treatment of malaria [188]. Surprisingly and against all odds, in the case of malaria, despite the fact that the parasites mitochondria are a therapeutic target, there is little investigation of the potential secondary effects of antibiotic therapies on the human mitochondrial genome and function [196]. Mitochondria-targeted pathogen products and the mitochondrial pathways affected by them provide potential novel targets for the rational design of drugs. Pathogen products may alter oxidative balance, mitochondrial transition pore permeability, mitochondrial membrane potential, electron transport chain, and ATP production [216]. 

Mitochondrial changes associated with bacterial infections and their antibiotic treatments most likely occur in the same manner in children and adults. However, some infections prevail during childhood and mitochondrial features may differ in children vs. adults. We have focused our review on those bacterial and parasitic infections presenting higher incidence rates in children, considering the most relevant characteristics of such infections in this population group. For each infection described in this review, a summary of the main mitochondrial assessments conducted in children presenting with the specific bacterial or parasitic infection and/or the antibiotic treatment, has been provided. It should be noted that there is a lack of studies on mitochondrial changes related to other pediatric infectious processes, herein not included, for which scarce data are available in children (e.g., regarding infection with *Neisseria gonorrhoeae*, *Clostridium botulinum*, *Streptococcus pneumoniae*, and *Clostridium tetani*). 

In many cases, the number of children infected and receiving drug therapy against a given infection is increasing. In addition, it is likely that if treatment is given as indicated in pregnant women with acute infections, the number of treatment-exposed newborns will also increase. Since studies and information are limited, especially in pediatric populations, it is essential to accurately assess the potential mitochondrial toxicity of such pharmacologic therapeutic options in a population as susceptible as newborns and infants. Remarkably, this has been studied in bacteriostatic antibiotics, which do not stimulate ROS production, suggesting that only bactericidal antibiotics result in major production of ROS [139]. Thus, mitochondrial dysfunction and oxidative damage induced by bactericidal antibiotics in mammalian cells may be alleviated by antioxidants or prevented by preferential use of bacteriostatic antibiotics [139]. 

In the near future, the identification of pathways or metabolites that are common to multiple pathogens remains an important challenge. Additionally, metabolic alterations that are directly involved in pathogen replication and not just a consequence of the infection need to be identified. More data and, in particular, longitudinal follow-up studies will be needed to contribute to the rather complex interaction of pathogens and treatments with mitochondrial metabolism [209], especially in children. 

## 6. Conclusions

Infants are an especially susceptible population group, particularly vulnerable during specific infective processes. Mitochondria play a major role in specific infections, due to: (i) molecular and/or ultrastructural alterations directly caused by the pathogen, (ii) molecular and/or ultrastructural changes caused by the treatments, (iii) their role as a therapeutic target in the disease, (iv) their implication in further clinical manifestations, (v) their identification as key targets of the infection process, (vi) their high adaptability during the infection process, and (vii) their protective role during the infection process. There is an urgent need to carry out longitudinal studies monitoring the long-term effects of bacterial and parasitic infections, which target mitochondria in developing children to further our understanding of these diseases.

## 7. Selection Criteria and Outcomes

We searched for scientific publications in three main database sources including Pubmed (MEDLINE), Web of Science, and SCOPUS. We included the common search terms: “mitochondria AND pediatric OR childhood OR infant OR children” for all the infectious diseases. For each infectious disease, we added the following terms: tuberculosis (TB), *Mycobacterium tuberculosis*; enterobacteria and enteroviruses, *Enterobacter cloacae*, *Proteus mirabilis*, *Escherichia coli* and *Salmonella enterica*; *Staphylococcus aureus*; meningitis, *Neisseria meningitidis and* *Streptococcus pneumoniae*; AND malaria, *Plasmodium falciparum*. We searched papers published in English between 1984 and 2020. We used the Rayyan QCRI software for systematic reviews (http://rayyan.qcri.org (accessed on 20 May 2020)), a free web and mobile app, that helps expedite the initial screening of abstracts and titles using a process of semi-automation while incorporating a high level of usability [217]. The studies were assessed for relevance to the topics and selected by two authors independently. With respect to the inclusion criteria, all randomized controlled studies in human models were included, as well as case reports and review articles. Animal models were excluded for this review.

## Figures and Tables

**Table 1:** Clinical differences in children vs. adults.

TB	Children	Adults
Cause	A complication of the pathophysiologic events surrounding the initial infection	A reactivation of organisms that were lodged in the apices of the lungs during hematogenous dissemination at the time of primary infection
Incubation period	Often only weeks to months	Often long (years to decades)
Pulmonary and extrapulmonary TB	Children are more prone to extrapulmonary disease but rarely develop contagious pulmonary disease	Adults are more prone to developing contagious pulmonary disease
Pulmonary location	Anywhere (25% multilobar)	Apical
Adenopathy	Usual	Rare (except HIV related)
Cavitation	Rare (except adolescents)	Common
Signs and symptoms	Unspecific	Typical

**Table 2:** *Mtb* molecules of direct or indirect mitochondrial interactions.

Name	Characteristics	Mechanism of Action
**Cpn60.2**	*Mtb* chaperone protein	Essential for bacterial growth Interacts with host mortalin (a mitochondrial protein that protects cells from apoptosis and is over-expressed in cancer cells). It has a strong anti-apoptotic activity dependent on its interaction with mitochondrial mortalin, for promoting *Mtb* survival [46]
**LpqH**	*Mtb* protein	Triggers an intrinsic or mitochondrial apoptosis pathway, with the participation of CytC and the AIF, a previously unrecognized mechanism [45]
**ESAT 6**	*Mtb* protein	A potent immunomodulator, which can induce cytolysis by disrupting mitochondrial membrane bilayers [47]
**Cyclophilin D (CypD)**	Mitochondrial matrix protein	Plays a key role in necrosis by regulating the PTP Its pharmacological inhibition in human macrophages leads to the inhibition of necrosis and reduction in *Mtb* growth A critical checkpoint of T cell metabolism for regulating disease tolerance in TB [40]

**Table 3:** Anti-TB first-line treatments, their mechanism of action, related mitochondrial dysfunction, and derived clinical adverse events.

Anti-TB Family	Characteristics	Mechanism of Action	Mitochondrial Dysfunction	Clinical Secondary Effects	Pediatric Studies
**Isoniazid** **(INH)**	Bacteriostatic It enters the mycobacterial cell by passive diffusion. Once inside, it acts as a prodrug and is activated by a mycobacterial enzyme, namely, KatG. Subsequent to its activation, the generated metabolite produces ROS [50]	It blocks the biosynthesis of mycolic acids [51]	Mitochondrial free radical generation Loss of mitochondrial Δψm Affects mitochondrial PTP Altered mitochondrial dynamics Impairs mitochondrial biogenesis [52]	It promotes liver injury mediated by a toxic reactive intermediate, acetylhydrazine, 2 weeks to 6 months after the initial treatment [53] Sometimes related to psychiatric manifestations [54]	Improving adherence to INH among children is vital in the fight to reduce world-wide TB mortality, especially in endemic areas with diagnostic difficulties [55] Quantification of the magnitude of INH-resistant TB and variation in frequency of INH resistance associated mutations is important [56]
**Rifampin** **(RIF)**	Bactericide from the rifamycin group Metabolism is mainly hepatic with 13–24% of the drug excreted unchanged in the urine [57]	Inhibits the essential *rpoB* gene product b-subunit of DNA-dependent RNA polymerase activity, acting early in transcription. Binding of the drug suppresses chain formation in RNA synthesis [57]	Mitochondrial free radical generation Loss of mitochondrial Δψm Affects mitochondrial PTP Altered mitochondrial dynamics [52]	Induces many drug metabolizing enzymes such as CYP1A2, 2C9, 2C19, and 3A4 and therefore increases chances of liver injury caused by additional anti-TB drugs [53] Hepatoxicity is initially characterized by an increase in plasma AST and ALT activities as well as bilirubin levels within 1–6 weeks of initiation [53]	Among children under the age of 18 years, treatment for 4 months had similar rates of safety and efficacy but a better rate of adherence than 9 months of treatment with INH [58] Pediatric patients were significantly more likely to complete latent TB infection treatment using RIF than with a 9-month INH regimen [59]
**Ethambutol (EMB)**	Bacteriostatic, although it also shows bactericidal effects if the concentrations are high enough Is active only in bacteria in the active multiplication phase [60,61]	Inhibits arabinosyl transferases involved in cell-wall biosynthesis [61,62]	Triggers disruption of mitochondrial CIV activity through a copper-chelating action [63,64,65,66] Loss of mitochondrial Δψm Fragmentation, disturbed calcium homeostasis, and the accumulation of abnormal intracellular vacuoles [66,67]	Optic neuropathy Hepatotoxicity Pruritus Joint pain Gastrointestinal upset and abdominal pain Malaise Headache Dizziness Psychiatric alterations such as mental confusion, disorientation, and hallucinations [62]	For children aged 5 years or more, EMB can be recommended at a dosage of 20 mg/kg/day for routine treatment, without taking more precautions than for adults; this should be included in official recommendations. For younger children, EMB can also be used without undue fear of side effects [68,69]
**Pyrazynamide (PZA)**	Fundamentally bacteriostatic, although it can also act as a bactericide [70]	A prodrug that needs to be activated to its active form by PZA (whose mutation generates bacterial resistance) It inhibits the FAS I system in the synthesis of mycolic bacteria mycolic acid Activated in an acidic medium, at the tubercular necrotic edges in which the inflammatory cells produce lactic acid [70]	Translocation of CytC from mitochondria to the cytosol, and consequent induction of the apoptotic pathway [71]	One of the most hepatotoxic tuberculostatics	The absorption and the clearance of PZA is slower, the elimination half-life longer and the volume of distribution higher in children compared with the reported values in the adult population [72] PZA is distributed uniformly in the body and serum levels are related to body weight, and a dose of 30 mg/kg bodyweight is appropriate in children [73] Lowering of PZA dosage is suggested in children for better patient compliance along with reduction in cost, side effects and toxicity without compromising its efficacy [74]

ALT, alanine aminotransferase; Anti-TB, antituberculosis; ARV, antiretrovirals; AST, aspartate aminotransferase; CI, complex I; CIII, complex III; CIV, complex IV; CytC, cytochrome C; DNA, deoxyribonucleic acid; EMB, ethambutol; ETC, electron transport chain; FAS, fatty acid synthetase; HIV/AIDS, human immunodeficiency virus infection and acquired immune deficiency syndrome; INH, isoniazid; KatG, catalase-peroxidase; NADH, nicotinamide adenine dinucleotide hydrogen; PI, protease inhibitors; PTP, permeability transition pore; PZA, pyrazynamide; RIF, rifampin; RNA, ribonucleic acid; ROS, reactive oxygen species; rpoB, beta subunit of RNA polymerase; TB, tuberculosis; Δψm, mitochondrial transmembrane potential.

**Table 4:** Antibiotic therapy of *S. aureus* infection, mechanism of action, associated mitochondrial damage, and relevant pediatric studies.

Drug	Antibiotic Type	Mechanism of Action	Mitochondrial Damage	Mitochondrial Interactions and Pediatric Studies
**Gentamicin**	Aminoglycoside	Bactericide Penetrates the bacteria and binds to the 30S and 50S ribosomal subunits, inhibiting protein synthesis [108]	Increases lactate production and inhibits Δψm [119] Induces mitochondrial ROS causing DNA damage [120,121]	Aminoglycoside antibiotics, in particular gentamicin and tobramycin, are still used in pediatric clinical practice. Acute kidney injury may occur in between 20% and 33% of children exposed to aminoglycosides. Cytoplasmic aminoglycoside then acts both directly and indirectly on the mitochondria, activating the intrinsic pathway of apoptosis via CytC, which, in turn, leads to the disruption of electron transport and ATP production and the formation of ROS [122]
**Linezolid**	2-oxazolidone	Bacteriostatic Inhibits bacterial protein synthesis by binding to 23S rRNA in the large ribosomal subunit and preventing the fusion of 30S and 50S ribosomal subunits and the formation of the translation initiation complex [123,124]	Inhibition of protein synthesis in mitochondria [123,124,125] Decrease in mitochondria-derived CIV impairs cellular OXPHOS and increases glycolysis [126] Deficient CV activity, which may be additive or synergistic in contributing to mitochondrial dysfunction and production of lactic acidosis [123,124,126,127] Mitochondrial dysfunction may lead to dysregulation of insulin secretion from pancreatic beta-cells, causing systemic hypoglycemia [125]	Linezolid-associated lactic acidosis by means of depressed mitochondrial CIV activity. Linezolid administration has been associated with lactic acidosis in adults; however, the same phenomenon has not been reported in children [125]
**Doxycycline**	Tetracycline	Bacteriostatic Inhibits bacterial protein synthesis by binding to the 30S ribosomal subunit [127]	Disrupts mitochondrial functions by decreasing Δψm and mitochondrial respiration. Decreases levels of ATP and the elevated levels of mitochondrial superoxide, intracellular ROS, protein carbonylation, and lipid peroxidation [128] Inhibits mitochondrial protein synthesis by reducing oxygen and increasing glucose consumption [129]	Doxycycline is a tetracycline-class antimicrobial for children >8 years of age for many common childhood infections. Doxycycline is not labeled for children ≤8 years of age, due to the association between tetracycline-class antibiotics and tooth staining, although doxycycline may be used off-label in severe conditions [130]
**Tigecycline**	Tetracycline	Bacteriostatic Inhibits protein translation in bacteria by binding to the 30S ribosomal subunit [131]	Inhibits mitochondrial OXPHOS Inhibits mitochondrial translation possibly by interacting with mitochondrial ribosome Deceases in mitochondrial respiration is the primary effect, while increased glycolysis flux is the secondary effect Induces mitochondrial ROS [132,133]	N/A
**Vancomycin**	Glycopeptide	Bactericide Inhibits biosynthesis of the bacterial cell wall, interferes with RNA synthesis, and damages the bacterial cell membrane [134]	Inhibits CI activity Causes apoptotic cell death by enhancing mitochondrial superoxide production leading to mitochondrial membrane depolarization followed by the caspase activities [135]	Higher vancomycin use does not improve outcomes in pediatric healthcare-associated *S. aureus* bacteremia but is associated with increased nephrotoxicity [136]. Vancomycin dosing strategies in pediatric patients should consider age and weight as well as renal function and indication [137].

ATP, adenosine triphosphate; CI, complex I; CIV, complex IV; CV, V complex; CytC, cytochrome C; DNA, deoxyribonucleic acid; OXPHOS, oxidative phosphorylation system; PTP, permeability transition pore; RNA, ribonucleic acid; ROS, reactive oxygen species; rRNA, ribosomal ribonucleic acid; *S. aureus, Staphylococcus aureus* and Δψm, mitochondrial transmembrane potential. Note: rifampin is also a therapeutic option that has not been included in Table 4, since it has been considered in Table 3. Quinolones, cotrimoxazole, and teicoplanin are also common treatments of pediatric TB but have not been included in this table due to limited information on their mitochondrial effects.

**Table 5:** Different causal pathogens of meningitis.

Meningitis Type		Microorganisms Responsible
**Bacterial meningitis**	In newborns and young infants (<3 months)	Group B *Streptococcus*
		*E. coli*
		*Listeria monocytogenes*
	In infants (>3 months) and children	*Hemophilus influenzae* type b *
		*Neisseria meningitidis*
		*Streptococcus pneumoniae*
	Others	Syphilis
		TB
**Viral meningitis**		Poliovirus *
		Enterovirus (e.g., coxsackie virus and echovirus)
		Mumps (paramyxovirus) *
		Herpes Simplex Virus (HSV)
**Other organisms that can also cause meningitis**		*Borrelia burgdorferi* (Lyme disease)
		Fungi such as candida, *Aspergillus*, or *Cryptococcus neoformans* in immunosuppressed patients

* uncommon due to vaccination campaigns.

**Table 6:** Antibiotic therapy against bacterial meningitis infection and associated mitochondrial effects.

Drug	Antibiotic Type	Mechanism of Action	Mitochondrial Damage	Pediatric Studies
**Ampicillin**	Betalactamic	Bactericide Inhibits the synthesis and repair of the bacterial wall [168]	Promotes ROS overproduction [138]	Ampicillin is a “broad-spectrum penicillin” used as therapy for suspected bacterial meningitis. Penetrates the blood–brain barrier sufficiently with an adequate dose and inflamed meninges. This drug revolutionized the treatment of bacterial meningitis in children [169,170]
**Cefotaxime**	Cephalosporin (also Betalactamic)	Bactericide Inhibits bacterial cell wall synthesis [171]	Apoptosis [172].	High-dose cefotaxime, while safe, is not reliably sufficient therapy for cephalosporin-non-susceptible pneumococcal meningitis, and combination therapy is recommended in children [173]
**Ceftriaxone**		Bactericide Broad spectrum and long acting Inhibition of cell wall synthesis [174]	Decreases Δψm [175] Reduces Ca^2+^ influx [176]	Ceftriaxone, widely used in children in the treatment of sepsis, is not reliably sufficient therapy for cephalosporin-non-susceptible pneumococcal meningitis [177]

Ca^2+^, calcium ion; CytC, cytochrome C; DNA, deoxyribonucleic acid; ROS, reactive oxygen species and Δψm, mitochondrial transmembrane potential.

**Table 7:** Antimalarial treatment in children and associated mitochondrial effects.

Type	Parasite and Resistance	Drug	Parasite’s Mitochondria Involvement
**Uncomplicated malaria (no signs of severity)**	*P. falciparum* or chloroquine-resistant strains	Atovaquone/proguanil	Inhibits the cyt bc1 complex, a key mitochondrial enzyme that catalyzes the transfer of electrons that maintain the membrane potential of the parasite’s mitochondria [188,190]
		Quinine sulfate + clindamycin or doxycycline (>8 years old)	Quinine sulfate affects parasitized erythrocytes and has a schizonticidal action [198,199]
		derivatives of artemisin, in Spain dihydroartemisin + piperaquine	Acts by depolarizing the mitochondrial membrane Increases production of ROS to alter mitochondrial functions ETC has some interactions with artemisinin, and probably plays an important role in its activation [200]
		Mefloquine	Blood schizonticide Breaks down hemoglobin in a food vacuole, producing a free heme pool and increasing ROS [201]
	*P. vivax, ovale, malariae*, or *falciparum* from chloroquine-sensitive area	Chloroquine	Blocks detoxification of heme, a by-product of hemoglobin degradation. During the asexual intraerythrocytic-stages, the parasite imports host cell hemoglobin into its food vacuole. Proteases in the food vacuole degrade hemoglobin into free amino acids, which are utilized in various growth processes. Heme is released during hemoglobin digestion and is essential for parasite growth as a cofactor for cytochromes in the parasite’s ETC [200]
		Primaquine	Eradicates hypnozoites that remain quiescent in the liver and prevents relapse [202]
**Severe malaria**	Normally caused by *P. falciparum*	Quinine gluconate IV diluted in glucose 5% + clindamycin IV (>8 years old)	Quinine sulfate affects parasitized erythrocytes and has a schizonticidal action [198,199]
		Artesunate	Is a semisynthetic analogue of artemisinin [203] Disrupts redox homeostasis in parasites [204,205] Increases ROS [206]

ETC, electron transport chain; ROS, reactive oxygen species.

## Abbreviations

AIF	Apoptosis-Inducing Factor
ANT	Adenine Nucleotide Translocase
APAF-1	Apoptosis Protease-Activating Factor-1
ATP	Adenosine Triphosphate
ADP	Adenosine Diphosphate
EMB	Ethambutol
ER	Endoplasmic Reticulum
ETC	Electron Transport Chain
CoQ	Coenzyme Q
CytC	Cytochrome C
DNAse	Deoxyribonuclease
FADH	Flavin and Adenine Dinucleotide Hydrogen
FMN	Flavin Mononucleotide
Fp	Flavoprotein
CI	Complex I
CII	Complex II
CIII	Complex III
CIV	Complex IV
CV	Complex V
COX	Cytochrome C Oxidase
CypD	Cyclophilin D
ESAT-6	Early Secretory Antigenic Target
ETC	Electron Transport Chain
HIV	Human Immunodeficiency Virus
HSV	Herpes Simplex Virus
IGRA	Interferon-Gamma Release Assays
IFNγ	Interferon-γ
IMM	Inner Mitochondrial Membrane
INH	Isoniazid
iNOS	Inducible Nitric Oxide Synthase
KCN	Potassium Cyanide
LOS	Lipooligosaccharide
MRC	Mitochondrial Respiratory Chain
MRSA	Methicillin Resistant *Staphylococcus Aureus*
*Mtb*	*Mycobacterium tuberculosis*
mtDNA	Mitochondrial Deoxyribonucleic Acid
mtRNA	Mitochondrial Ribonucleic Acid
NADH	Nicotinamide Adenine Dinucleotide Hydrogen
NHBA	Neisseria Heparin-Binding Antigen
NO	Nitric Oxide
OMM	Outer Mitochondrial Membrane
OXPHOS	Oxidative Phosphorylation System
PARP	Poly ADP-Ribose Polymerase
Pi	Inorganic Phosphate
PTP	Permeability Transition Pore
PZA	Pyrazinamide
RIF	Rifampin
ROS	Reactive Oxygen Species
rRNA	Ribosomal Ribonucleic Acid
SOD	Superoxide Dismutase
TB	Tuberculosis
TCA	Tricarboxylic Acid
TNFα	Tumor Necrosis Factor-α
TNT	TB Necrotizing Toxin
tRNA	Transfer Ribonucleic Acid
TST	Tuberculin Skin Test
Δψm	Mitochondrial Membrane Potential
